# Prediction CH_4_ Emissions from the Wetlands in the Sanjiang Plain of Northeastern China in the 21^st^ Century

**DOI:** 10.1371/journal.pone.0158872

**Published:** 2016-07-13

**Authors:** Tingting Li, Qing Zhang, Wen Zhang, Guocheng Wang, Yanyu Lu, Lijun Yu, Ran Zhang

**Affiliations:** 1 LAPC, Institute of Atmospheric Physics, Chinese Academy of Sciences, Beijing, 100029, China; 2 Anhui Climate Center, Hefei, 230031, China; 3 CCRC, Institute of Atmospheric Physics, Chinese Academy of Sciences, Beijing, 100029, China; Yonsei University, REPUBLIC OF KOREA

## Abstract

The Sanjiang Plain has been experienced significant wetland loss due to expanded agricultural activities, and will be potentially restored by the China National Wetland Conservation Action Plan (NWCP) in future. The objective of this study is to evaluate the impact of future climate warming and wetland restoration on wetland CH_4_ emissions in northeast China. We used an atmosphere-vegetation interaction model (AVIM2) to drive a modified biogeophysical model (CH4MOD_wetland_), and projected CH_4_ flux variations from the Sanjiang Plain wetlands under different Representative Concentration Pathway scenarios throughout the 21^st^ century. Model validation showed that the regressions between the observed and simulated CH_4_ fluxes by the modified model produced an R^2^ of 0.49 with a slope of 0.87 (p<0.001, n = 237). According to the AVIM2 simulation, the net primary productivity of the Sanjiang Plain wetlands will increase by 38.2 g m^-2^ yr^-1^, 116.6 g m^-2^ yr^-1^ and 250.4 g m^-2^ yr^-1^ under RCP 2.6, RCP 4.5 and RCP 8.5, respectively, by the end of this century. For RCP 2.6, 4.5 and 8.5 scenarios, the CH_4_ fluxes will increase by 5.7 g m^-2^ yr^-1^, 57.5 g m^-2^ yr^-1^ and 112.2 g m^-2^ yr^-1^. Combined with the wetland restoration, the regional emissions will increase by 0.18‒1.52 Tg. The CH_4_ emissions will be stimulated by climate change and wetland restoration. Regional wetland restoration planning should be directed against different climate scenarios in order to suppress methane emissions.

## Introduction

Methane (CH_4_) is a very efficient greenhouse gas, with a Global Warming Potential of 25 on a 100-year time horizon, and is currently the second anthropogenic greenhouse gas after CO_2_ [1]. The radiative forcing of CH_4_ has been modified from 0.48 W m^-2^ [1] to 0.97 W m^-2^ [2, 3] when its indirect global warming effect was incorporated. Natural wetlands are the largest natural source in the present-day global CH_4_ budgets. They have emitted 100–231 Tg CH_4_ yr^-1^, which accounts for 20–39% of the annual global CH_4_ emission [1, 4]. In addition, the CH_4_ emission from wetlands increased by 7% from 2003 to 2007 [5].

CH_4_ emissions from wetlands are controlled by climatic conditions [6, 7]. The two major climate factors that controls methane emissions from wetlands are temperature and precipitation. The temperature can influence the microbial process rates of microbial CH_4_ production [6, 8, 9], and the precipitation can determine the water table depth which determines saturated and unsaturated proportion of the wetland [10–12]. Over the twenty-first century, projected climate warming is expected to increase rates of heterotrophic respiration, yet plant primary production (NPP) could increase due to the CO_2_ fertilization [13, 14]. The increased NPP would stimulate CH_4_ emissions since it is the main source of the methanogenic substrat [15,16].

Increased atmospheric CO_2_ due to anthropogenic emissions is expected to lead to significant climate change in the 21st century [1, 2, 17]. A new set of scenarios, e.g., the representative concentration pathways (RCPs) was designed in the IPCC fifth assessment report (AR5) based on the fifth phase of the Coupled Model Intercomparison Project5 (CMIP5) [18, 19]. RCPs represent classes of mitigation scenarios that produce emission pathways following various assumed policy decisions that would influence the time evolution of the future emissions of GHGs, aerosols, ozone, and land use/land cover changes [20]. Significant warming was simulated under RCPs for IPCC AR5 during the 21st century [21–23]. The world’s temperature by 2100 is projected to increase by 1.3–1.9°C under RCP 2.6, 2.0–3.0°C under RCP 4.5 and 4.0–6.1°C under RCP 8.5 [23]. In China, the trend of climate warming and wetting will also intensify in the future [24, 25]. The warming tendency from 2011 to 2100 is 0.06°C per decade for RCP 2.6, 0.24°C per decade for RC P4.5, and 0.63°C per decade for RCP 8.5 [24]. The national mean precipitation will increase, and the increasing precipitation in the northern regions is significant and greater than in the southern regions in China [24].

The Sanjiang Plain, located in Heilongjiang province in the northeast China, was formerly the largest wetland complex in China [26, 27]. Site experiments indicated that freshwater marshes in the Sanjiang Plain had a relative high CH_4_ fluxes compared with other reions, e.g., the Qinghai Tibet Plateau [28, 29] and other ecosystems, e.g., rice paddies [30]. The climate change and CO_2_ fertilization must have great impacts on CH_4_ emissions on the Sanjiang Plain in the 21^st^ century.

Moreover, the northeast China contributes more than 50% of the CH_4_ sources from the natural wetlands on a national scale [31–33]. The area in the Sanjiang Plain has decreased significantly and deteriorated in quality owing to expanded agricultural activities since the 1950s [26,34–36]. In order to reverse this trend, wetlands restoration projects must be implemented in future [37]. Since the Ramsar Convention on Wetlands in 1971, wetland conservation (maintenance and sustainable use) and restoration (recovery of degraded natural wetlands) have been high priorities for many countries. The Chinese government also announced a project named “the China National Wetland Conservation Action Plan (NWCP)” in 2000 and approved it in 2003 [38]. During 1980–2005, the cumulative area of wetland reserves has increased to 500 km^2^ in China [39–41]. At the provincial level, the Heilongjiang province did an excellent work in wetland reservation and restoration [42]. The government of Heilongjiang Province enacted several laws and accompanying policies to ensure wetland protection. They provided legal support for wetland protection and restoration [37]. For example, they published the three versions of “Regulations of Heilongjiang Province on the protection of wetlands” in 2003, 2010 and 2016, respectively. Compared with the period of 1950–2000, the decrease rate of the wetland area in Heilongjiang province has been greatly reduced after the year 2000 [43]. Over all, assessing the trends of CH_4_ emissions under different climate scenarios and the wetland restoration policies in the Sanjiang Plain is important to the government for the greenhouse gas management. However, this is still a knowledge gap in the current studies.

Models provide a powerful tool to predict long-term CH_4_ dynamics under climate change. The models include empirical models and process-based models. Empirical CH_4_ emission models have been developed by directly correlating the observed CH_4_ fluxes to controlling factors, such as standing water depth, soil temperature, plant primary productivity or ecosystem productivity [10, 15, 44]. However, these empirical relationships cannot be extrapolated to other sites where the soil and climate conditions are different from the experimental sites. Compared with the empirical models, they can simulate CH_4_ emissions with different degrees of complexity and are integrated with other processes [45, 46]. We established a process-based model named CH4MOD_wetland_, which was generally capable of simulating the seasonal and interannual variations in CH_4_ emissions from different wetland sites in China and North America [46]. The objective of the research was using this model to make predictions of CH_4_ emissions from the wetland of the Sanjiang Plain under the climate scenarios of IPCC AR5.

## Materials and Methods

### Study Area

**Fig 1:**
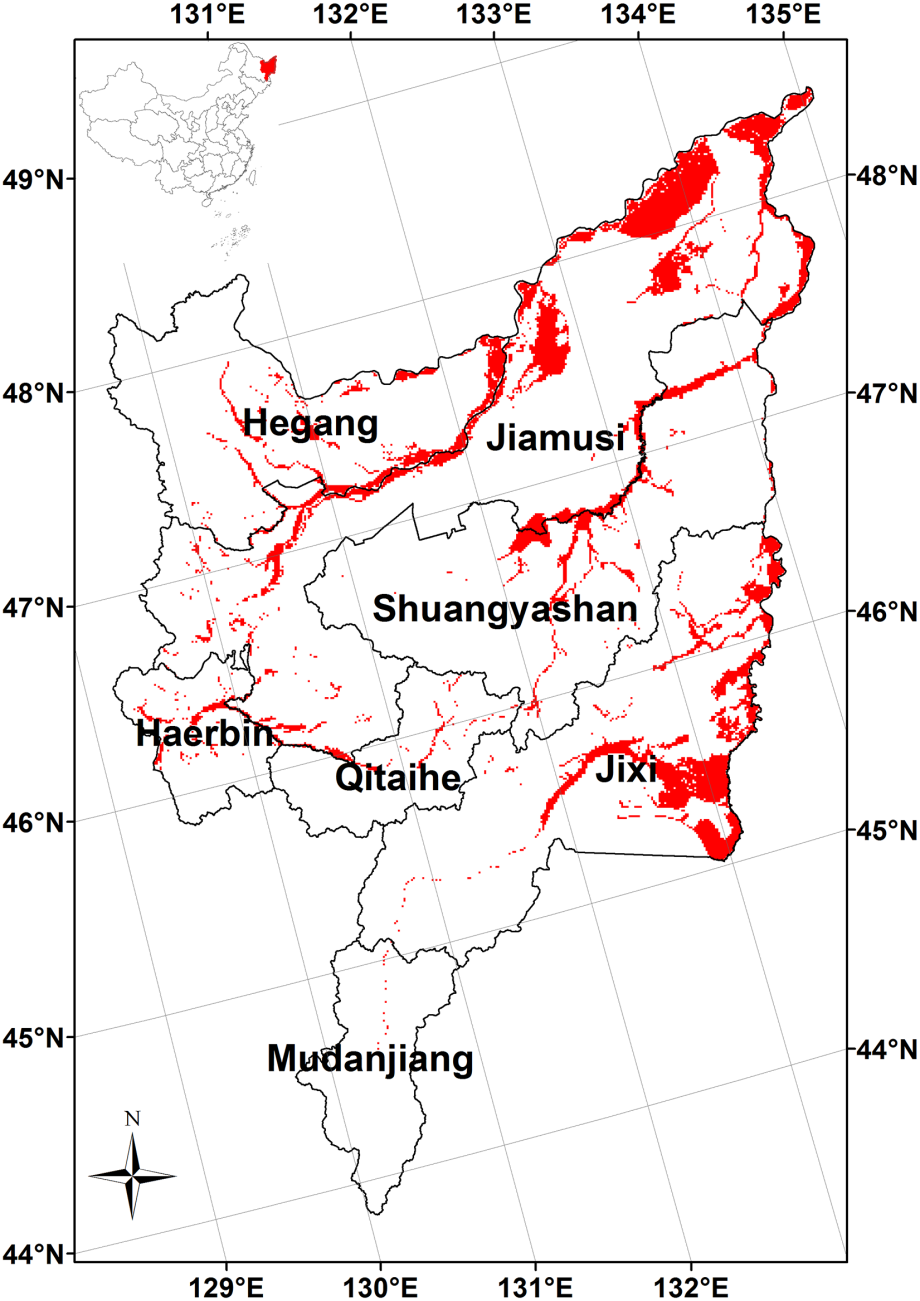
Spatial distribution of the wetland in the Sanjiang Plain. Map constructed in ESRI ArcMAP 10.1. Base image of the boundaries is obtained from the Open Platform of Ministry of Civil Affairs of the People’s Republic of China [48]. The wetland distribution is from [43].

The Sanjiang Plain is located in the eastern part of Heilongjiang Province, northeast China (Fig 1). It is located between 43°50'N and 48°28'N latitudinally and between 129°11'E and 135°05'E longitudinally. It covers a total area of 108900 km^2^ [47], of which 10700 km^2^ is natural wetland at present [43]. The Sanjiang Plain covers 7 cities (Fig 1), including 23 counties. The policies related to wetlands conservation and restoration are made by the city’s forestry bureau.

The average above sea level of the Sanjiang Plain is 56 m. The climate in this area belongs to the temperate humid and subhumid continental monsoon climate. The annual mean precipitation is around 600 mm and the annual mean air temperature is ~1.9°C. The average evapotranspiration from the natural wetlands ranges from 540 to 580 mm. The types of vegetation vary from *Deyeucia angustifolia* to *Carex lasiocarpa* as the standing water depth increases. The above-ground net primary productivity ranges from 260 to 700 g m^-2^ [49–54]. The wetland area is mainly vegetated with *Deyeuxia angustifolia* and *Carex lasiocarpa* plants, which account for about 20% and 80% of the vegetation in the Sanjiang Plain, respectively [55].

### Model Framework

**Fig 2:**
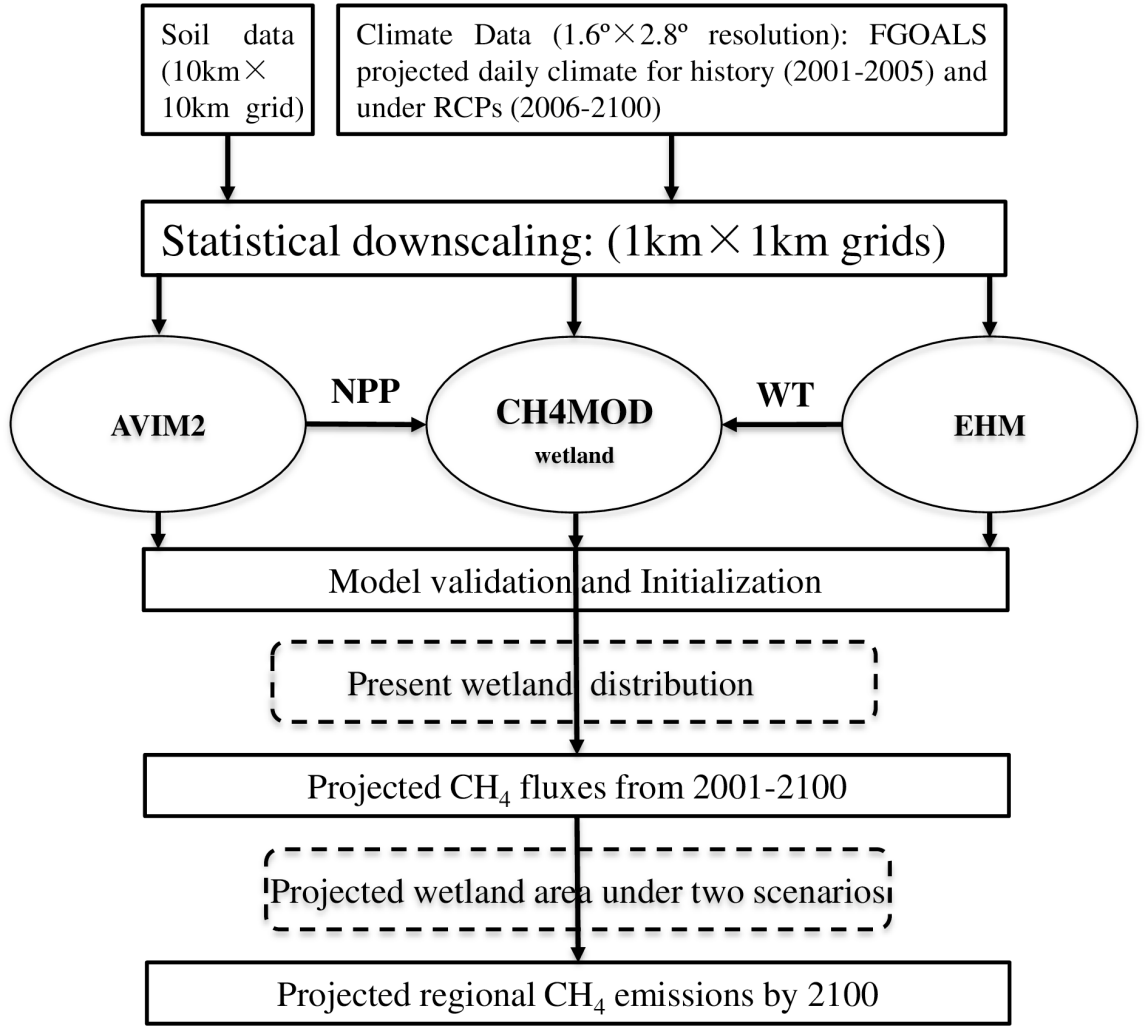
Model framework for simulating CH_4_ emissions from the Sanjiang Plain from 2001–2100 under RCPs. CH4MOD_wetland_ is a biogeophysical model to simulate CH_4_ fluxes from natural wetlands. AVIM2 (Atmospheric-Vegetation Interaction Model, version 2) is a process-based terrestrial ecosystem model that simulates seasonal and interannual variations in biophysical and biogeochemical processes at the land surface. EHM is an empirical hyrological model to simulate daily water table depth.

In this study, we used an integrated modeling framework centered on a biogeophysical model named CH4MOD_wetland_ to simulate CH_4_ emissions from the wetlands in the Sanjiang Plain. Fig 2 shows the framework for the simulation study.

CH4MOD_wetland_ is a biogeophysical process-based model that is developed to describe the processes of CH_4_ production, oxidation and emission from natural freshwater wetlands [32, 36, 46]. The model adopted the hypothesis of CH4MOD model, which is used to simulate CH_4_ emissions from rice paddies [56, 57], and made modifications according to the difference between the natural wetlands and rice paddies. In CH4MOD_wetland_, we focused on the different supply of methanogenic substrates between natural wetlands and rice paddies. The methanogenic substrates were derived from the root exudates, the decomposition of plant litter and the soil organic matter. Methane production rates were determined by the methanogenic substrates and the influence of environmental factors including soil temperature, soil texture and soil Eh. Inputs to the CH4MOD_wetland_ include daily air temperature, daily water table depth, soil texture, soil organic matter and the annual above-ground net primary productivity (*ANPP*). The outputs are daily CH_4_ emissions. Previous studies give the detailed description of model calibration and the parameter values [36, 46].

Previous studies showed that the model was generally capable of simulating the seasonal and interannual variations in CH_4_ emissions from different wetland sites in China and North America [46]. However, the insufficiency still exists in the model, e.g., the dynamics of the water table and the *ANPP* limits its upscaling to a long-term and a regional scale.

In the model framework we used an empirical hydrological model (EHM) [58], which can simulate daily water table depth to drive the CH4MOD_wetland_ (Fig 2). The EHM simulates the balance between the water input (such as precipitation and surface inflow, etc.) and water output (such as evapotranspiration and runoff, etc.). Water table dynamics (*ΔWT*, in cm) are determined directly by the balance between the water input (*Sin*, cm), runoff (*F*_*out*_, cm) and evapotranspiration (*ET*, cm). No runoff occurs during the period of freezing temperatures from November to March:

$$\Delta WT=\{\begin{array}{ll} S_{in}-F_{out}-ET & \left(Apr\sim Oct\right)\\ P-ET & \left(Nov\sim Mar\right) \end{array}$$ (1)

$$WT_{i}=WT_{i-1}+\Delta WT$$ (2)

$$S_{in}=\alpha_{0}\times P$$ (3)

$$F_{out}\;=\{\begin{array}{ll} a_{1}\times \left(WT_{i}-D_{1}\right)+a_{2}\times \left(WT_{i}-D_{2}\right)\; & \left(WT_{i}>D_{1}\right)\\ a_{2}\times \left(WT_{i}-D_{2}\right) & \left(WT_{\text{i}}>D_{2}\right)\\ 0 & \left(WT_{i}<D_{2}\right) \end{array}$$ (4)

where *WT*_*i*_ represents the daily water table. According to Zhang [57], *S*_*in*_ is a function of precipitation (*P*) (Eq 3), and *F*_*out*_ includes surface outflow and ground outflow, both of which are determined by the water table [58] (Eq 4). *D*_*1*_ and *D*_*2*_ represent two critical levels (cm) of *WT*. The experimental constants (*α*_*0*_, *a*_*1*_, *a*_*2*_, *D*_*1*_, *D*_*2*_) in Eqs (3) and (4) are experimental constants which should be calibrated by trial and error method (Table 1).

**Table 1:** Parameter values for the main kinds of wetland in the Sanjiang Plain.

Wetland Plant Type	*α*_*0*_	*a*_*1*_	*a*_*2*_	*D*_*1*_ (cm)	*D*_*2*_ (cm)
***Deyeuxia angustifolia***	1.1	0.012	0.02	0	-15
***Carex lasiocarpa***	1.25	0.011	0.005	0	-15

The Priestley-Taylor model was used to calculate *ET* [59–61]. The net radiation (*R*_*n*_), which is used to calculate *ET* in the Priestley-Taylor model, was calculated using the equations of the modified Penmann-Monteith model [62]. When the water table position value was less than zero, the water table depth (*WT*) in CH4MOD_wetland_ was considered to be zero.

The annual *ANPP* is an important input of CH4MOD_wetland_. In CH4MOD_wetland_, it is used to calculate the root exudates, which is the main substrate for methanogens. In the model framework, we used the atmospheric-vegetation interaction model (AVIM2) model to calculate the annual *ANPP* to drive CH4MOD_wetland_ (Fig 2). AVIM2 is a dynamic terrestrial biosphere model with independent intellectual property and international recognition [63]. AVIM2 model consists of three sub-models: terrestrial physical module of soil-vegetation-atmosphere transformation (SVAT), vegetation physiological growth module and soil organic matter (SOM) module. The net primary productivity (*NPP*) is the residue of gross canopy photosynthesis minus maintenance and growth respiration. The AVIM2 model has been widely used to predict the national *NPP* change under different climate environment in China [64, 65]. We used the proportion of above-ground and total *NPP* to calculate the *ANPP* [46]. More details about the model structure, model inputs, outputs and parameters were described in previous studies [63, 64].

Three steps were needed for the model simulation. Firstly, model validation is important to test the performance of the model framework (Fig 2). The observations from the Sanjiang Mire Wetland Experimental Station, Chinese Academy of Sciences were used for model validation. We used the historical climate dataset (described in the next section) in the grid where the site located to drive the model framework and compared the simulated annual *ANPP*, daily water table depth and daily CH_4_ fluxes with the observations. Secondly, we ran the EHM and AVIM2 for 200 years to the equilibrium status and obtained the initial water table depth. Table 2 shows the model experimental design in this study. At last, we ran the model framework from 2001 to 2100. The simulated CH_4_ fluxes from 2001 to 2010 were used as the baseline simulations.

**Table 2:** Detail description of the model experimental design.

Model	Simulation	Period	Outputs	Driving data	Source
**EHM**	Model validation	2002–2005	Daily *WT*	Daily climate (*T*, *T*_*max*_, *T*_*min*_, *P*, *SP*, *SH*)	FGOALS outputs (2002–2005) FGOALS outputs (1991–2000) FGOALS outputs (2001–2005 & 2006–2100)^#^
	Model initialization^&^	1991–2000^&^	Initial *WT*		
	Model projection	2001–2100	Projected daily *WT*		
**AVIM2**	Model validation	2002–2005	Annual *ANPP*	Daily climate (*T*, *Pr*, *u*, *v*, *SH*); Soil texture; Annual CO_2_ concentration	FGOALS outputs (2002–2005); ISSCAS
	Model initialization^&^	1991–2000^&^	Initial status		FGOALS outputs (1991–2000); ISSCAS
	Model projection	2001–2100	Projected annual *ANPP*		FGOALS outputs (2001–2005 & 2006–2100); ISSCAS
**CH4MOD**_**wetland**_	Model validation	2002–2005	Daily CH_4_ fluxes	Daily climate (*T*); Soil (*SAND*, *SOM*, *BD*); Daily *WT*, Annual *ANPP*	FGOALS outputs (2002–2005); ISSCAS; Outputs of EHM and AVIM2
	Model projection	2001–2100	Projected CH_4_ fluxes		FGOALS outputs (2001–2005 & 2006–2100); ISSCAS; Outputs of EHM and AVIM2

^&^ Model initialization used the climate data of 1991–2000 to drive the model and run 200 years to equilibrium status.

^#^ Climate data before 2006 are the historical outputs by FOGALS; Climate data after 2006 are the projections by FGOALS under RCPs.

EHM is Empirical hydrological model; *WT* is water table depth; *ANPP* is above ground biomass; *T* is air temperature; *T*_*max*_ is maximum air temperature; *T*_*min*_ is minimum air temperature; *P* is precipitation; *SP* is surface pressure; *SH* is specific humidity; *u* is meridional wind; *v* is zonal wind; *SAND* is soil sand fraction; *SOM* is soil organic matter; *BD* is bulk density.

The projected gridded CH_4_ emissions were the product of the projected CH_4_ fluxes and the wetland area on a grid scale. The regional CH_4_ emissions were the summation of the gridded CH_4_ emissions. In this study, two scenarios of the projected wetland area were assumed: the projected wetland area will remain the present level or be restored according to the NWCP by 2100.

### Data Sets

The data sets used for model site-specific validation were from the observations. The Sanjiang Mire Wetland Experimental Station, Chinese Academy of Sciences is located in Tongjiang city, Heilongjiang province, China, at approximately 47°135'N, 133°131'E. Methane emissions in the *Deyeucia angustifolia* marsh (M-D) and *Carex lasiocarpa* marsh (M-C) were measured approximately twice per week by using a closed opaque chamber [66] from April to the early-October in 2002–2005. The mixing ratios of CH_4_ were detected by gas chromatography (Agilent 4890) with flame ionization detection [67]. The standing water depth was recorded synchronously with the measurement of CH_4_ emissions, but was not measured from early-April to late-June, as well as during the entire growing season of 2002 for M-C and M-D, respectively. The annual *ANPP* for M-D and M-C was measured three times per month during 2002–2005 [49].

The driving datasets for model validation, model initialization and model prediction including climate daily datasets as well as soil data are listed in Table 2. The climatic datasets are the outputs from FGOALS [68–70], which were provided by the State Key Laboratory of Numerical Modeling for Atmospheric Sciences and Geophysical Fluid Dynamics (LASG), the Institute of Atmospheric Physics (IAP), the Chinese Academy of Sciences (CAS). The FGOALS simulated daily climate for history (1850–2005) and for the RCP scenarios (2006–2100) in CMIP5. The history climate data were used to drive the model framework for model validation and initialization. For the first half baseline period (2001–2005), the history climate data were also used to drive the model framework. The climate under RCP scenarios were used for model projection from 2006 to 2100. Three RCP scenarios were used in this study. They have been derived from integrated assessment models (IAM) [21]. RCP 8.5 is a “high pathway” for which radiative forcing reaches >8.5 W m^-2^ by 2100 and continues to rise for some amount of time. It has an approximate CO_2_ equivalent concentration of 1370 ppm in 2100. RCP 4.5 is an intermediate “stabilization pathways” in which radiative forcing is stabilized at approximately 4.5 W m^-2^ after 2100. The approximate CO_2_ equavalent concentrations is 650 ppm. RCP 2.6 is a pathway where radiative forcing peaks at approximately 3 W m^-2^ before 2100 and then declines 2.6 W m^-2^ in 2100. The peak approximate CO_2_ equavalent concentration reaches 490 ppm before 2100 under RCP 2.6. These scenarios include time paths for emissions and concentrations of the full suite of greenhouse gases (GHGs) and aerosols and chemically active gases, as well as land use/land cover. The original resolution of the climate datasets is 2.8°×1.6°(longitude×latitude). We statistically downscaled [71] the datasets to a resolution of 1 km×1 km.

The soil characteristic data (10 km×10 km) were developed by the Institute of Soil Sciences, Chinese Academy of Sciences. We also downscaled the data into 1 km×1 km.

The map of Sanjiang region boundary and the prefecture-level city boundaries was obtained from the open platform of Ministry of Civil Affairs of the People’s Republic of China [48] (Fig 1). The wetland distribution (Fig 1), which was used to calculate the area-weighted CH_4_ fluxes as well as the regional CH_4_ emissions was from the remote sensing data with the resolution of 1km×1km [43]. The recent research reported that the wetland area in the Sanjiang Plain was 10714 km^2^ in 2008 [43]. This is considered as the first scenario that the projected wetland area will remain 10714 km^2^ by 2100. The second scenario is called the wetland restoration scenario. According to the NWCP, 14000 km^2^ of the natural wetlands will be recreated from 2004 to 2030, accounting for approximately 7.5% of the current area of natural wetlands in China [32, 43]. The wetland area will increase by 25% by 2100 in the light of this restoration rate. This restoration will be mainly controlled by the government policies. We assumed that the wetland restoration would be averaged distributed in the wetland region, and simply calculated the projected regional CH_4_ emissions by this fraction under the wetland restoration scenario.

## Results and Discussion

### Model Validation

**Fig 3:**
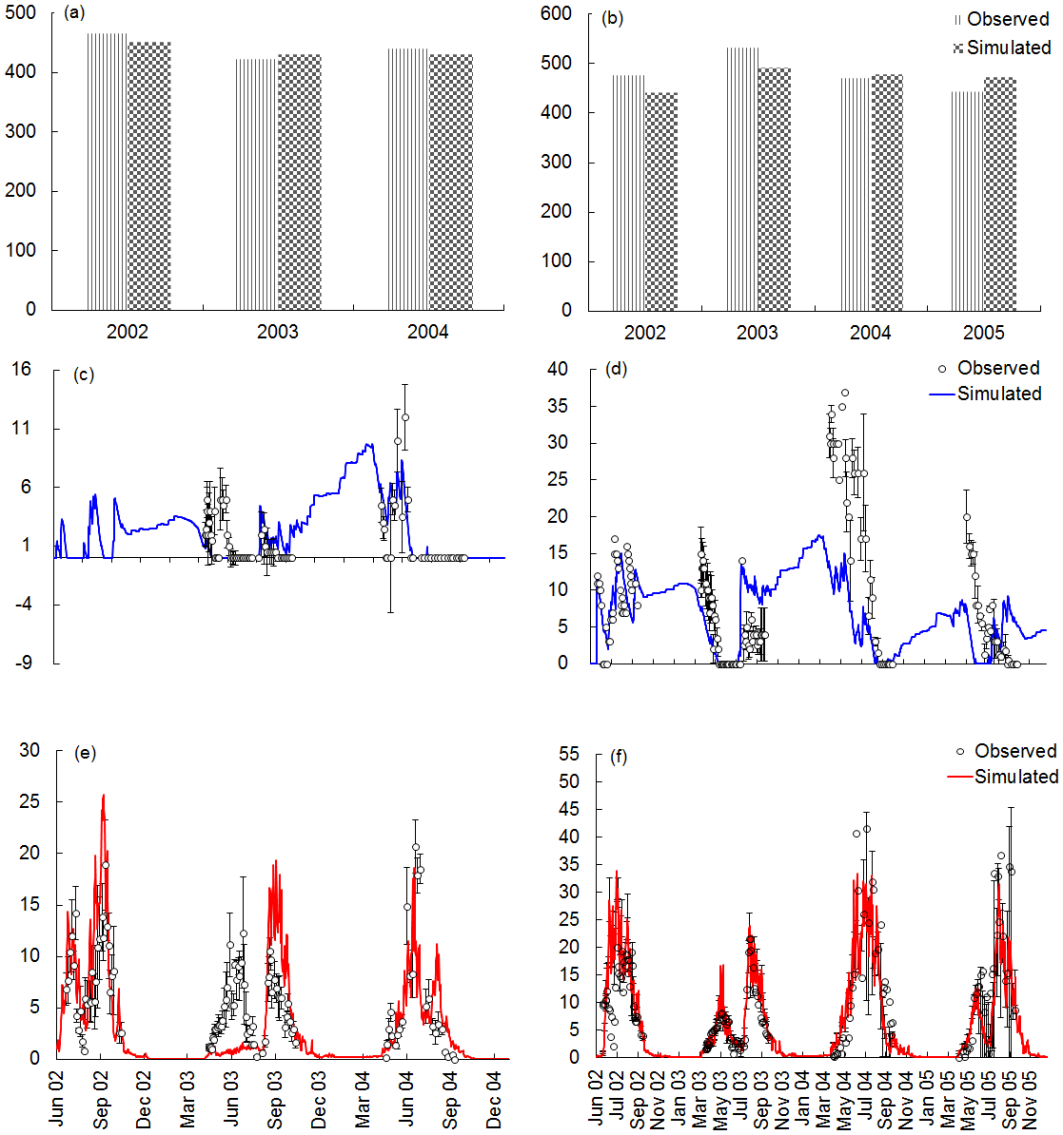
Comparison of simulated and observed annual aboveground net primary productivity by AVIM2. (a) for M-D site, (b) for M-C site. Comparison of simulated and observed seasonal patterns of water table depth and CH_4_ fluxes on the Sanjiang Plain by EHM and CH4MOD_wetland_. (c) Water table depth and (e) CH_4_ flux for M-D site; (d) Water table depth and (f) CH_4_ flux for M-C site. The vertical bars are standard deviations from 3 sampling replicates.

Fig 3 shows the annual *ANPP* and the seasonal patterns of the simulated and observed standing water depth and CH_4_ emissions in M-D site (Fig 3a, 3c and 3e) and in the M-C site (Fig 3b, 3d and 3f) on the Sanjiang plain. The simulated annual *ANPP* generally agreed well with the measured data in the M-D site (Fig 3a) and the M-C site (Fig 3b). For the M-D site, the AVIM2 model overestimated *ANPP* by 2% in 2003, and underestimated *ANPP* by 3% and 2% in 2002 and 2004, respectively (Fig 3a). More significant differences between the simulated and observed *ANPP* are shown at M-C site, with ~7% underestimation for the first two years and 2%—7% overestimation for the last two years, respectively (Fig 3b). Overall, the discrepancies are 2%—7% between the observed and simulated *ANPP*.

The model can basically simulate the seasonal variations in standing water depth (Fig 3c and 3d) and CH_4_ fluxes (Fig 3e and 3f). However, clear differences between the simulations and observations are shown in 2003 for the water table depth (Fig 3c) and the CH_4_ fluxes (Fig 3e) for the M-D site. During April to July 2003, the empirical hydrological model can’t simulate the flooded situation. The simulated water table depth was always zero (Fig 3c). This inaccurate simulation of water table depth induced a significant negative discrepancy of CH_4_ fluxes between the modeled and observed CH_4_ fluxes. When the water table depth drops to zero, the anaerobic environment will become aerobic environment, which will greatly decrease CH_4_ emissions [11]. In contrast, there’s a positive discrepancy occurred during the period from September to October 2003 between the modeled and observed CH_4_ emissions from the M-D site (Fig 3e). This is mainly because the overestimated water table depth from this period (Fig 3c). There was no standing water after the early September. However, the EHM simulated a higher water table depth (Fig 3c), which induced lower soil redox potential and accumulated CH_4_ emissions (Fig 3e).

For the M-C site, the EHM underestimated the water table depth from April to July of 2004 and 2005 (Fig 3d). However, the simulated CH_4_ flux matches the observed flux well during the same period (Fig 3f). This correspondence occurred because in CH4MOD_wetland_, CH_4_ emissions are not sensitive to the standing water depth when it is aboveground for a period [46], which is in agreement with the observation of [72] that the soil redox potential decreases to a certain limit and maintains that level when the standing water depth is above the soil surface for a given amount of time.

**Fig 4:**
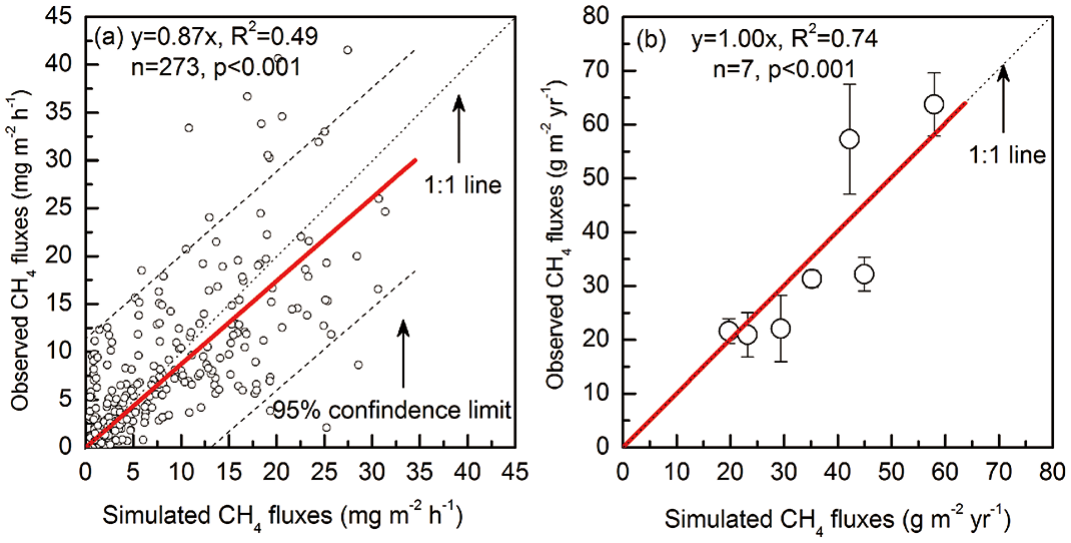
Observed vs. simulated CH_4_ emissions from M-D site (June 2002– December 2004) and M-C site (June 2002– December 2005) on the Sanjiang Plain. (a) CH_4_ fluxes, dashed lines are 95% confidence limits. (b) Total amount of annual/seasonal CH_4_ emissions, dotted line is 1:1 line, the vertical bars are standard deviations from 3 sampling replicates.

Fig 4 shows the observed and simulated CH_4_ emissions from the Sanjiang Plain. Using 273 datasets, regression of the observed versus simulated CH_4_ fluxes produced an R^2^ of 0.49 with a slope of 0.87 (p<0.001) (Fig 4a). The performance of CH4MOD_wetland_ was good for the total annual/seasonal CH_4_ amounts (Fig 4b). Regression of the computed and observed annual CH_4_ amounts yielded an R^2^ of 0.74 with a slope of 1.00 (n = 7, p<0.001) (Fig 4b).

### Projected Climate Changes and *ANPP* under RCP Scenarios

**Fig 5:**
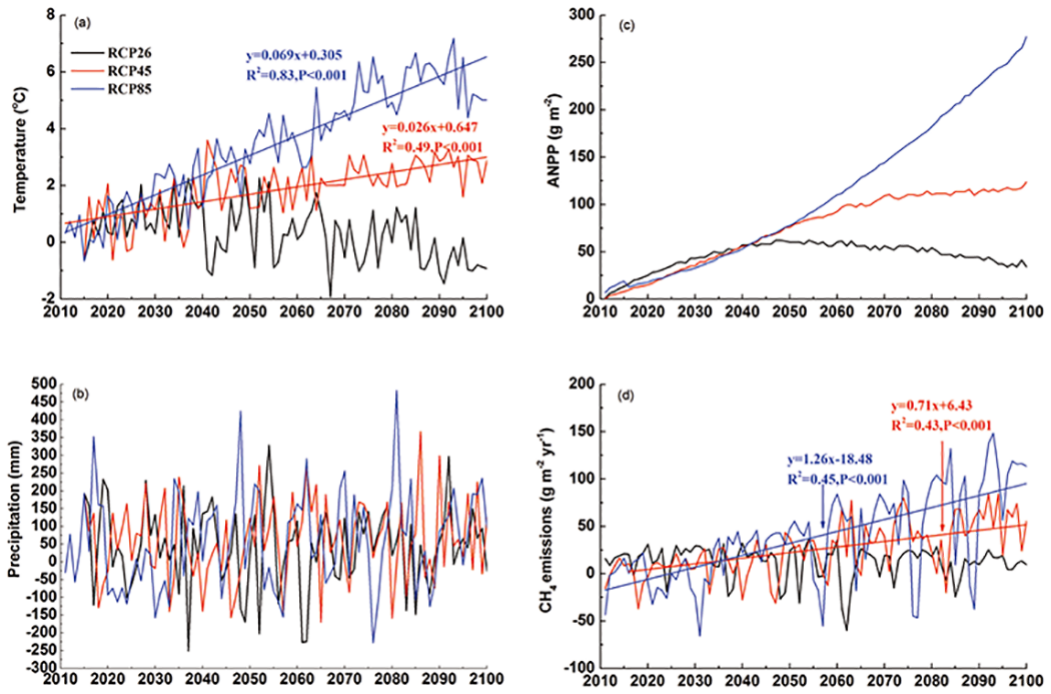
Projected area-weighted annual mean air temperature (a) and annual precipitation (b) by FGOALS, annual aboveground net primary productivity (c) by AVIM2 and CH_4_ fluxes (d) by CH4MOD_wetland_ from the wetlands of the Sanjiang Plain for RCP 2.6 (black line), RCP 4.5 (red line) and RCP 8.5 (blue line) relative to the average 2000s, respectively.

Fig 1 and Table 3 shows the projected climate for the 2011–2100 by FGOALS under the RCP scenarios. The climate under RCP 4.5 and RCP 8.5 scenarios shows a warmer and wetter trend compared with the period 2001–2010. There is a significant warming tendency for the period from 2011 to 2100 for RCP 4.5 and RCP 8.5, with an increase rate of 0.26 and 0.69°C per decade (p<0.001) (Fig 5a). Compared with period of 2000s, the annual area-weighted air temperature will increase by 2.73°C and 5.42°C for RCP 4.5 and RCP 8.5 until 2100 (Table 3). For RCP 2.6 scenario, the annual area-weighted air temperature increased significantly during the middle term (2041–2070), and then decrease during the long term (2071–2100) (Fig 5a and Table 3).

**Table 3:** Air temperature, precipitation, *ANPP* and CH_4_ emissions compared with the average value of 2001–2010 under RCP scenarios over the Sanjiang Plain.

Periods	RCP 2.6				RCP 4.5				RCP 8.5			
	*T*	*P*	*ANPP*	CH_4_	*T*	*P*	*ANPP*	CH_4_	*T*	*P*	*ANPP*	CH_4_
**2011–2020**	0.40	75.3	14.6	9.1	0.79	21.2	8.8	-2.2	0.35	81.6	14.7	2.2
**2021–2030**	0.81	35.7	35.9	9.3	0.69	78.5	27.3	10.2	1.29	-57.0	26.5	-11.0
**2031–2040**	1.30	24.7	50.0	5.6	1.13	43.3	46.7	8.2	2.14	54.9	44.1	11.5
**2041–2050**	0.37	21.5	59.2	2.4	2.34	5.0	66.7	12.5	2.80	103.5	67.4	36.8
**2051–2060**	0.45	65.2	59.2	7.3	1.76	78.5	85.7	21.3	3.75	37.3	95.0	31.8
**2061–2070**	0.40	-6.5	56.4	-8.0	2.07	96.7	101.2	38.0	3.91	96.9	128.7	52.4
**2071–2080**	0.01	70.9	53.8	8.9	2.47	65.1	109.7	44.6	5.38	35.5	165.1	48.9
**2081–2090**	0.05	0.8	46.1	-2.0	2.54	74.5	112.5	40.8	5.86	69.6	206.9	66.1
**2091–2100**	-0.75	81.1	38.2	5.7	2.73	76.6	116.6	57.5	5.42	133.0	250.4	112.2

The annual area-weighted precipitation over the Sanjiang Plain will continue to increase in the future (Fig 5b and Table 3). No significant linear increase was shown in the annual area-weighted precipitation under the RCP scenarios. The annual mean precipitation shows great interannual variation (Fig 5b). Compared with the period 2000s, the annual mean precipitation will increase by 81.1 mm, 76.6 mm and 133.0 mm until 2100 under RCP 2.6, RCP 4.5 and RCP 8.5 scenarios (Table 3).

Estimated by the AVIM2 model, the annual area-weighted *ANPP* is expected to increase by the CO_2_ fertilization. Under RCP 8.5 scenario, the *ANPP* will increase by approximately 250 g m^-2^ yr^-1^ by the end of this century (Table 3, Fig 5c). The *ANPP* will increase by 85.7 g m^-2^ yr^-1^ by 2050s under RCP 4.5, and then the increase rate will decline. Until 2100, the *ANPP* will increase by 116.6 g m^-2^ yr^-1^ under this scenario (Table 3, Fig 5c). For RCP 2.6 scenario, the annual area-weighted *ANPP* will increase rapidly up to mid-century and then begin to decline (Table 3, Fig 5c).

Conclusions on the effect of climate change on global *NPP* are inconsistent. The *NPP* could both increase [14, 73–75] and decrease [65, 76, 77] under future climate scenario. This study indicated that the *NPP* would increase by 7%, 28% and 63% under RCP 2.6, RCP 4.5 and RCP 8.5 scenarios by 2100. Jin [78] predicted the similar increases in *NPP* by 2%, 32% and 50% under RCP 2.6, RCP 4.5 and RCP 8.5 scenarios by 2100 in the wetlands of the Qinghai Tibet Plateau, respectively. The result in this study is also consistent with the result of Wieder [14], who reported that the global *NPP* will increase by 63±27% under RCP 8.5 scenario, if the nitrogen and phosphorus limitation was not considered. The results of this study under RCP 4.5 is slightly higher than the prediction by Melillo [73], who reported that the global *NPP* will increase by 20–26%, but lower than the predictions by Ji [64], who reported that the national *NPP* would increase by 36% under the CO_2_ concentration of 620 ppm in China.

### Spatial Temporal Changes in CH_4_ Fluxes under RCP Scenarios by 2100

Table 3 and Fig 5d provides the projected changes in the area-weighted annual mean CH_4_ fluxes from 2011 to 2100 compared with the average value of 2000s under RCP 2.6, RCP 4.5 and RCP 8.5 in the Sanjiang Plain, respectively. In the near term of 2030s, the increments in area-weighted annual mean CH_4_ fluxes are around 10 g m^-2^ yr^-1^ for all scenarios (Table 3). However, in the middle term (2061–2070), and the long term (2090–2100), the area-weighted CH_4_ fluxes will continue to increase under RCP 4.5 and RCP 8.5 scenario, but maintain stability under RCP 2.6 scenario (Table 3). By the end of this scenario, the area-weighted CH_4_ fluxes will increase by 57.5 g m^-2^ yr^-1^ and 112.2 g m^-2^ yr^-1^, which are 74% and 147% of the 2000s under RCP 4.5 and RCP 8.5 scenario, respectively (Table 3).

**Fig 6:**
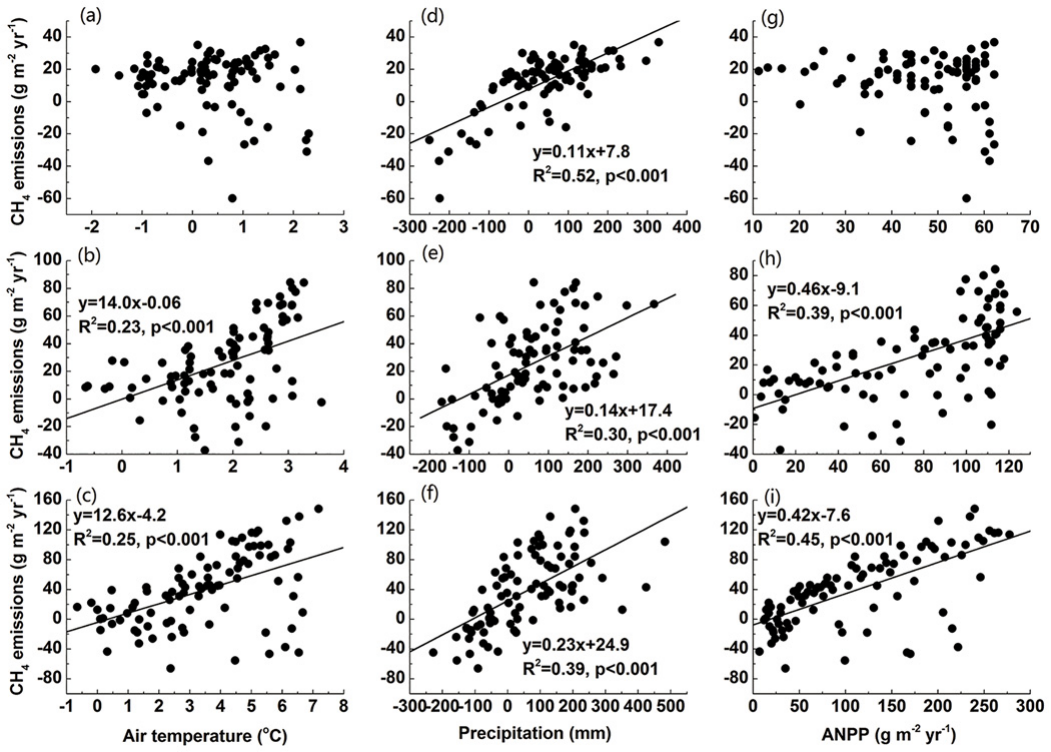
Regression between air temperature, annual precipitation and aboveground net primary productivity and CH_4_ fluxes. (a), (b) and (c) for RCP 2.6; (d), (e) and (f) for RCP 4.5; (g), (h) and (i) for RCP 8.5.

The projected CH_4_ fluxes by CH4MOD_wetland_ show a significant increase under RCP 4.5 and RCP 8.5 scenarios, with increasing rates of 0.71 g m^-2^ yr^-1^ and 1.26 g m^-2^ yr^-1^, respectively (Fig 5d, p<0.001). These annual increases are strongly and positively correlated with the increases of air temperature (Fig 6d and 6g), precipitation (Fig 6e and 6h) and the *ANPP* (Fig 6f and 6i) (p<0.001). This indicated that both the climate and *ANPP* will promote CH_4_ fluxes under RCP 4.5 and RCP 8.5 scenario by the end of 21^st^ century. For RCP 2.6 scenario, the increase of CH_4_ fluxes displays a significant positive correlation with the precipitation (Fig 6b, p<0.001), which indicates that the wetter climate will accelerate CH_4_ emissions in future. However, a weak negative correlation (Fig 6a, p = 0.17) between the air temperature and CH_4_ fluxes implies the warmer climate may be not the main factor to promote CH_4_ emissions.

Under RCP 2.6 scenario, highest increment in CH_4_ fluxes with a value of 37.6% is projected in the MuDanJiang city, followed by 29.3% in HaErBin city (Table 4). Lowest increment is projected to happen in the JiXi city (Table 4). However, there are different patterns under RCP 4.5 and RCP 8.5 scenarios. Under RCP 4.5 scenario, the JiXi city and MuDanJiang city show a higher projected increase in CH_4_ fluxes, approximate two times of other cities (Table 4). The HaErBin city, HeGang city and JiaMuSi city are projected to have the highest increases under RCP 8.5, which is over 150% compared with the present value (Table 4).

**Table 4:** Spatial change of CH_4_ emissions compared with the average value of 2000s under RCP scenarios.

City	RCP 2.6	RCP 4.5	RCP 8.5
**HaErBin**	29.3%	61.4%	150.9%
**HeGang**	23.3%	62.3%	150.4%
**JiaMuSi**	23.9%	61.9%	150.6%
**JiXi**	10.9%	117.7%	137.9%
**MuDanJiang**	37.6%	118.0%	124.8%
**QiTaiHe**	22.3%	63.0%	149.8%
**ShuangYaShan**	17.4%	65.0%	145.2%

Previous studies show uncertainties in predicting CH_4_ fluxes under different climate conditions. This study predicted increases in CH_4_ fluxes of 8%, 76% and 147% by 2100 from the wetlands of the Sanjiang Plain under RCP 2.6, RCP 4.5 and RCP 8.5 scenarios. In the wetlands of the Qinghai Tibet Plateau, Jin [78] predicted the CH_4_ increases of 16%, 46% and 70% under RCP 2.6, RCP 4.5 and RCP 8.5 scenarios using TEM model. Our result is lower than Jin’s result under RCP 2.6, and higher than Jin’s result under RCP 4.5 and RCP 8.5. However, our result under RCP 8.5 is similar Zhuang’s result [79], who predicted that CH_4_ emissions from the wetlands in northern high latitudes would more than double over the century in a scenario of projected atmospheric CO_2_ mole fraction of approximately 1152 ppm by 2100. Moreover, the prediction in this study under RCP 4.5 is consistent with the result of Shindell [80], who predicted a rise of 78% in the global CH_4_ emissions under doubled CO_2_ scenario (approximate 700 ppm). Christensen also projected the increase in CH_4_ emissions (56%) under doubled CO_2_ scenario in the arctic tundra [81], which is slightly lower than our results.

### Impacts of Wetland Restoration on Regional CH_4_ Emissions in the Sanjiang Plain

If the wetland area maintains the level of 2008 of 10714 km^2^ [43], according to the result of this study (Table 3), the regional CH_4_ emissions will increase by 0.15 Tg, 0.58 Tg and 1.21 Tg by the end of this century under RCP 2.6, RCP 4.5 and RCP 8.5 scenarios, respectively. These increases of CH_4_ sources account for 1.4%, 5.5% and 11.6% of the CH_4_ budget from terrestrial ecosystems in China [82], respectively. If the emissions are converted to CO_2_-eq using the factors of 25 on a 100-year time scale, the increase of CH_4_ emissions will induce a positive global warming potential (GWP) of 3.65, 14.62 and 30.49 Tg CO_2_-eq yr^-1^. Under the wetland restoration scenario, the regional CH_4_ emissions will increase by 0.18, 0.73 and 1.52 Tg in the Sanjiang Plain by 2100 under RCP 2.6, RCP 4.5 and RCP 8.5 scenarios, respectively.

This study indicates that the wetland restoration will increase CH_4_ emissions. This conclusion is also found in previous studies [83–87]. According to the spatial changes in CH_4_ fluxes under different scenarios (Table 4), regional appropriate planning of the wetland restoration for the government may suppress the methane emission as less as possible. For the RCP 2.6 scenario, the wetland restoration should be firstly projected in JiXi city and ShuangYaShan city, followed by QiTaiHe city, JiaMuSi city and HeGang city in the Sanjiang Plain under limited funds. The MuDanJiang city should be the last city to restore wetlands. However, if“high pathway” happens (RCP 8.5), the MuDanJiang city should be considered as the first one to carry out the restoration. For the RCP 4.5 scenario in which radiative forcing is stabilized at approximately 4.5 W/m^2^ after 2100, the JiXi city and MuDanJiang city should not considered to be the prior regions to implement wetland restoration, so that less CH_4_ will be emitted to the atmosphere.

### Uncertainties and Future Needs

This study project future trends of the CH_4_ emissions from the natural wetlands in the Sanjiang Plain. The present result is still uncertain due to incomplete model structure, inaccurate of water table depth simulation, limited model calibration and validation as well as the bias in future climate projections.

Firstly, although the process-based model has become more specialized to adequately represent CH_4_ production, oxidation, and transport by considering various factors and controls, there are still knowledge gap in the model structure [88]. One example of an insufficiently modeled process is the influence of thawing permafrost on CH_4_ production in the arctic region. The Sanjiang Plain belongs to the freeze–thaw area. In the Sanjiang Plain, the freezing and thawing phase have 7–8 months and play important role in the greenhouse gases emission [89]. Measurements showed that there were significant CH_4_ and CO_2_ emission peak values during thawing time [89]. Lack of thawing, freezing and snow melting process will induce distorted CH_4_ simulation in winter and freeze-thaw period. In future, the observed effects of thawing and freezing of soils and snow melting on CH_4_ production and diffusion should be considered in the present model.

Secondly, CH_4_ fluxes are strongly controlled by the water table depth in the Sanjiang Plain [11]. The model sensitivities also show that water table depth is one of the most sensitive factors to CH_4_ emissions [46, 90, 91]. So without accurately simulated of water table positions, estimates of CH_4_ and are poorly constrained [92]. Take this study for example, the negative discrepancy from April to July 2003 at the M-D site was induced by the underestimated water table depth (Fig 3c and 3e). Several approaches have been adopted for obtained wetland water table depth dynamics. Spatially distributed hydrological models, e.g., FLATWOODS [93] and SWAT [94] predict spatial distributions of water table depth by using spatial databases of topography, climate, soil, and vegetation at a watershed scale. However, the detailed spatial databases are difficult to obtain. TOPMODEL scheme [95] based on the topographic wetness index (TWI) is widely used to model regional water table depth in natural wetland [91, 96, 97]. However, the TWI is static and relies on the assumption that local slope, tanβ, is an adequate proxy for the effective downslope hydraulic gradient which is not necessarily true in low relief terrain [98], such as the Sanjiang Plain. In this study, we used the empirical model because it needs fewer inputs and are suitable to small-scale studies. Overall, the simulation of spatial temporal water table dynamics is a big challenge in future studies.

Thirdly, the observational data related to processes of and controls on CH_4_ production, consumption, and transport are still limited, which induced insufficient model calibration and validation. Take this study for example, only CH_4_ emissions from two sites (M-D and M-C) were available. However, measurements of net CH_4_ emissions are only useful to constrain a few model parameters. The net CH_4_ emissions are the balance of CH_4_ production and oxidation. There’s no validation of the CH_4_ production, CH_4_ oxidation as well as the partition between diffusion, ebullition and plant transportation. More detailed observations are needed not only limited to the net CH_4_ fluxes in future, in order to make the biogeochemical model more accurate.

Last but not least, the bias in climate projections by FOGLAS would inevitably induced uncertainties in the CH_4_ projection results. The Global climate models (GCMs) are widely used for projections of future climate change. The GCMs can capture the large-scale features of climate, but more uncertainties appear at regional and smaller scales [99]. Uncertainties resulting from the multi-model ensemble were shown in the climate change projections in China [24, 100]. Previous studies [24] analyzed the projected climate change in China under RCPs based on 11 RCMs simulations. The results showed that the projected precipitation change was more credible in northeast China than other regions. However, a greater difference in the projected temperature changes between the RCMs was shown in the northeast China. In this study, only one GCM’s outputs were used as the driving factor, which were inevitably induced uncertainties to the model results. However, the daily step of the model framework limited the climate data acquisition, since most GCMs supply only monthly step outputs. In addition, we used a statistical downscale for the climate projections. This is a computationally efficient method based on a stable climate. Compared with the statistical downscale method, dynamical downscaling is implemented using a fine-scale regional climate model (RCM) with a better representation of local terrain that simulates climate processes over the region of interest [100]. However, to an ecosystem modeler, the high computational cost of the dynamical downscaling is too difficult. A publicly accessible dynamical downscaling database with a daily step would greatly benefit the research community in future studies [78].

## Conclusions

Using a biogeophysical model CH4MOD_wetland_ and an atmosphere-vegetation interaction model (AVIM2) model, this study investigated the CH_4_ fluxes from the wetlands in the Sanjiang Plain under different Representative Concentration Pathways scenarios during the 21st century. The model simulations at the site level were able to closely match the field-observed CH_4_ fluxes after integrating an empirical hydrological model. In response to future climate change and CO_2_ fertilization, both net primary productivity and CH_4_ fluxes will increase, especially under RCP 8.5 scenario. On a regional scale, highest increment in CH_4_ fluxes will happen in the southern Sanjiang Plain under RCP 2.6 and RCP 4.5 scenarios, while occur in the northeastern Sanjiang Plain under RCP 8.5 scenario. The “China National Wetland Conservation Action Plan (NWCP)” by the government would inevitably increase total CH_4_ emissions by 0.18, 0.73 and 1.52 Tg in the Sanjiang Plain by 2100 under RCP 2.6, RCP 4.5 and RCP 8.5 scenarios, respectively.
